# In-hospital contact investigation among health care workers after exposure to smear-negative tuberculosis

**DOI:** 10.1186/1745-6673-4-11

**Published:** 2009-06-08

**Authors:** Felix C Ringshausen, Stephan Schlösser, Albert Nienhaus, Anja Schablon, Gerhard Schultze-Werninghaus, Gernot Rohde

**Affiliations:** 1 Department of Medicine III – Pneumology, Allergology and Sleep Medicine, University Hospital Bergmannsheil, Bochum, Germany; 2 Department of Medicine, Spital Bülach, Bülach, Switzerland; 3 Department of Occupational Medicine, University Hospital Bergmannsheil, Bochum, Germany; 4 Department of Occupational Health Research, Institution for Statutory Accident Insurance and Prevention in Health and Welfare Services, Hamburg, Germany

## Abstract

**Background:**

Smear-negative pulmonary tuberculosis (TB) accounts for a considerable proportion of TB transmission, which especially endangers health care workers (HCW). Novel Mycobacterium-tuberculosis-specific interferon-γ release assays (IGRAs) may offer the chance to define the burden of TB in HCW more accurately than the Mantoux tuberculin skin test (TST), but the data that is available regarding their performance in tracing smear-negative TB in the low-incidence, in-hospital setting, is limited. We conducted a large-scale, in-hospital contact investigation among HCW of a German university hospital after exposure to a single case of extensive smear-negative, culture-positive TB with pulmonary involvement. The objective of the present study was to evaluate an IGRA in comparison to the TST and to identify risk factors for test positivity.

**Methods:**

Contacts were prospectively enrolled, evaluated using a standardized questionnaire, the IGRA QuantiFERON^®^-TB Gold in Tube (QFT-GIT) and the TST, and followed-up for two years. Active TB was ruled out by chest x-ray in QFT-GIT-positive subjects. Independent predictors of test positivity were established through the use of logistic regression analysis.

**Results:**

Out of the 143 subjects analyzed, 82 (57.3%) had close contact, but only four (2.8%) experienced cumulative exposure to the index case >40 hours. QFT-GIT results were positive in 13 subjects (9.1%), while TST results were positive in 40 subjects (28.0%) at an induration >5 mm. Overall agreement was poor between both tests (kappa = 0.15). Age was the only predictor of QFT-GIT-positivity (Odds ratio 2.7, 95% confidence interval 1.32–5.46), while TST-positivity was significantly related to Bacillus Calmette-Guérin vaccination and foreign origin. Logistic regression analysis showed no relation between test results and exposure. No secondary cases of active TB were detected over an observational period of two years.

**Conclusion:**

Our findings suggest a low contagiosity of the particular index case. The frequency of positive QFT-GIT results may in fact reflect the pre-existing prevalence of latent TB infection among the study population. TB transmission seems unlikely and contact tracing not generally warranted after cumulative exposure <40 hours. However, the substantially lower frequency of positive QFT-GIT results compared to the TST may contribute to enhanced TB control in health care.

## Background

Tuberculosis (TB) is a major cause of illness and death worldwide [1]. In contrast, Germany is a low-incidence country with steadily decreasing annual numbers of new TB infections (6.1 per 100,000 inhabitants in 2007) [2], where targeted testing of at-risk groups as well as diagnosis and treatment of latent TB infection (LTBI) in individuals with recent exposure are fundamental components of TB control strategies [3].

Although cross-reactivity following vaccination with Bacillus Calmette-Guérin (BCG) or exposure to non-tuberculous mycobacteria is common, the tuberculin skin test (TST) has been applied for the diagnosis of LTBI for about a century [4]. In-vitro interferon-γ release assays (IGRAs) that measure the amount of interferon-(IFN)-γ secreted by T-cell lymphocytes after stimulation with highly Mycobacterium-tuberculosis-(MTB)-specific antigens have been developed as alternative diagnostics. They are broadly recommended and increasingly used in contact investigations [5,6], as they provide distinct advantages over the TST. Their sensitivity for detecting active TB, which is commonly used as a surrogate for LTBI, is at least equal and their specificity is clearly superior, at least in populations that contain a proportion of BCG-vaccinated individuals, as they are not confounded by BCG vaccination. Moreover, they are appropriate for the serial testing of health care workers (HCW) as they avoid boosting of immune responses and possess distinct logistical conveniences [7-9].

Acid-fast bacilli smear-negative, culture-positive pulmonary TB accounts for a considerable proportion of TB transmission. In 2007, 56.3% of all infectious pulmonary TB cases reported to the responsible German authority (Robert Koch Institute) were smear-negative [2]. Although in general considered less contagious, smear-negative TB index cases were found to be responsible for 13–17% of TB transmission in molecular-epidemiologic studies [10,11]. The characteristics of smear-negative TB cases include prolonged contact, lack of isolation and delayed diagnosis and treatment, thus highlighting its impact as a nosocomial disease and its importance to TB control in high-income, low-incidence countries and health care.

TB contact investigations in the in-hospital setting are often challenging due to patient movement and the changing work assignment of personnel [12]. Particularly HCW are considered at risk for the occupational transmission of TB infection, even after brief exposure [13,14]. In this regard, IGRAs may offer the unique chance of defining the burden of TB in HCW more accurately [15].

**Figure 1:**
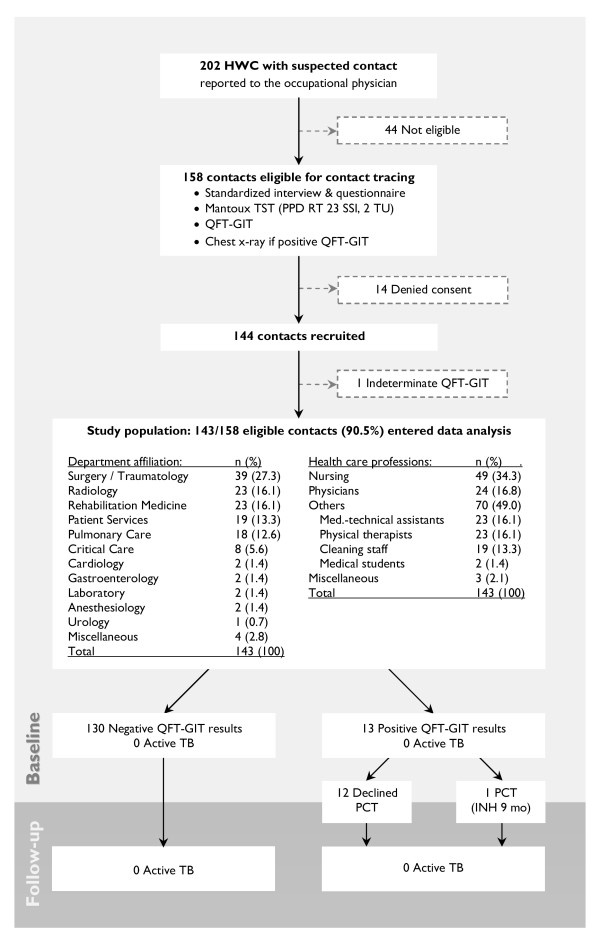
**Study profile**. HCW = health care workers; IGRA = interferon-γ release assay. PCT = preventive chemotherapy; PPD = purified protein derivate; QFT-GIT = QuantiFERON^®^-TB Gold in Tube; TST = tuberculin skin test.

We conducted an in-hospital contact investigation of a single index patient with extensive smear-negative, culture-positive TB including non-cavitary pulmonary involvement, who had a complicated in-hospital course of about three months and numerous contacts in various medical departments and disciplines (Figure 1, also see additional file 1: Definition of the index case). The aim of the present study was to compare the performance of the IGRA QuantiFERON^®^-TB Gold in Tube (QFT-GIT) with the Mantoux tuberculin skin test (TST) in a large-scale in-hospital contact investigation among German HCW after exposure to a single case of smear-negative, culture-positive pulmonary TB and to identify independent risk factors of test positivity.

## Methods

### Study design and subjects

We prospectively enrolled eligible HCW. The suspected time of in-hospital infectivity was 57 days from referral to our neurotraumatological department on January 10^th ^until March 7^th ^2007, when isolation and antimycobacterial treatment were initiated. Contacts were evaluated using a standardized interview and questionnaire, TST, IGRA and chest x-ray if IGRA results were positive. The diagnostic and therapeutic course of the index case throughout the entire hospital stay was reconstructed. A total of 202 HCW with suspected contact were reported to the responsible occupational physician. Inclusion criteria were an age of 18 years and above, actual contact to the index case during infectivity and written and informed consent. The study cohort was longitudinally observed regarding progression to active TB for a period of two years (mean 106 ± 1.5 weeks) until March 13^th ^2009. All HCW were informed of TB-related symptoms, instructed on self-monitoring and reporting and subject to routine follow-up screening according to German Occupational Safety and Health legislation. All QFT-GIT-positive subjects were radiologically followed up as recommended by national guidelines [6].

### Diagnostic methods

The TST was performed by the Mantoux method using 0.1 ml (two tuberculin units) of purified protein derivative (PPD) RT 23 (Statens Serum Institute, Copenhagen, Denmark). The test was administered strictly intradermally to the volar side of the forearm and was read 72 hours after application. The transverse diameter of induration was measured and documented as described previously [16]. A diameter of >5 mm was considered positive according to national guidelines [6]. Both the administration and the reading of the TST were performed by the same experienced occupational physician in order to minimize observer-dependent variation.

As an IGRA, the QFT-GIT (Cellestis, Carnegie, Australia) was used. ELISAs and the interpretation of QFT-GIT results were performed according to the manufacturer's instructions that consider a result positive if the IFN-γ response of TB antigen minus Nil was ≥ 0.35 IU/ml (see additional file 2: Addendum methods section). All assays met quality control standards. The occupational physician who read the TST was blinded to the QFT-GIT results determined by the laboratory team and vice versa. In participants with positive QFT-GIT results, active TB was ruled out by physical examination and chest x-ray, and the subsequent administration of preventive chemotherapy with Isoniazid (INH) for nine month was suggested following current national and international recommendations [3,6].

### Interview and questionnaire items

A standardized interview was conducted by the occupational physician. A questionnaire as well as medical records were used for the collection of demographic and clinical data with special attention paid to established individual risk factors regarding the acquisition of a new TB infection, the reactivation of LTBI or false negative or false positive TST results (see additional file 2: Addendum methods section) [3,6]. BCG vaccination status was reassured by medical and vaccination records or the presence of vaccination scars.

### Evaluation of exposure

Close contact and thus a relevant risk of transmission even after short exposure was assumed if there was exposure during airway management, transesophageal echocardiography, gastroscopy or face-to-face contact during a physical examination, physiotherapy or patient care and nursing (e. g. oral hygiene, patient transfer) [6]. The cumulative exposure time was calculated according to Arend et al. [17]: the contact period in weeks, the average number of days per week on which there had been contact, the number of contacts per shift according to work assignment and the average contact time (min) were multiplied and resulted in the cumulative exposure time (min). In order to achieve a maximum accuracy, special attention was paid both to the index patient's course throughout the different medical departments, and to the work assignments and working schedules of the HCW.

### Statistical analysis

Data analysis was performed using SPSS, version 11.5 (SPSS Inc., Chicago, Illinois). Categorical data were compared by Pearson's chi-squared or Fisher's exact test, where appropriate. Normal distribution in continuous variables was determined with the Kolmogorov-Smirnov test and differences were subsequently determined either with the student's t-test or the Mann-Whitney-U test. Spearman correlation coefficients and kappa values were calculated for both tests. Relations were described as odds ratio (OR) and 95% confidence interval (CI). ORs for test results depending on different putative predictive variables were calculated using logistic regression. Model building was performed backwards using the chance criteria for variable selection [18]. All p values reported were calculated two-sided with statistical significance set to p < 0.05. The study protocol was approved by the ethics committee of the Ruhr-University, Bochum. All study participants gave their written and informed consent.

## Results

### Study population

Between June and August 2007, mean 17 ± 2 weeks after last exposure to the source case, 202 HCW with suspected contact were evaluated. Of those, 44 had not had contact or had not been exposed during the time of infectivity. Of the 158 eligible contacts, 14 denied consent and 144 were recruited for the study. One subject (with a negative TST result) was excluded from data analysis due to an indeterminate QFT-GIT result (see additional file 3: Detailed description of the subject with indeterminate QFT-GIT result). Finally, 143 of the 158 eligible contacts (90.5%) constituted the study population (Figure 1). The demographic and clinical features of the study population are shown in Table 1. The HCWs' different affiliations and professions are displayed in Figure 1. The present population was characterized by a mean age of 38 ± 10 years (range 20–62) and a mean duration of employment in health care of 14 ± 10 years (range 1–42). As these variables were highly correlated (r = 0.72, p < 0.001), the latter was not considered for the logistic regression analysis. More than one half of the subjects were BCG vaccinated (51.0%), while only a small number of subjects had been born in a high endemic TB country (2.8%).

**Table 1:** Characteristics of the study population

Variables	n	%
Subjects, total	143	100
Sex		
Male	44	30.8
Female	99	69.2
Age categorized*		
18 to 39 years	84	58.7
40 to 49 years	36	25.2
≥ 50 years	23	16.1
Duration of employment in health care*		
1 to 5 years	35	24.5
6 to 10 years	25	17.5
11 to 20 years	50	35.0
21 to 42 years	33	23.1
Foreign country of birth^†^		
Yes	25	17.5
No	118	82.5
Birth in high burden country^‡^	4	2.8
BCG vaccination		
Yes	73	51.0
No	56	39.2
unknown	14	9.8
Cumulative exposure time		
≤ 1 hour	76	53.1
> 1 to 8 hours	42	29.4
> 8 to 40 hours	21	14.7
> 40 hours	4	2.8
Close contact	82	57.3
Prior TST	117	81.8
Positive prior TST result	45	38.5
TST >5 mm induration	40	28.0
TST >10 mm induration	28	19.6
Positive QFT-GIT result	13	9.1
Health care professions		
Nursing	49	34.3
Physician	24	16.8
Other	70	49.0
Affiliation with Pulmonary Care	18	12.6
Own history of TB	1	0.7
Family history of TB	8	5.6

* Age and duration of employment were highly correlated (r = 0.72, p < 0.001). ^† ^Mostly Poland (n = 9) and Turkey (n = 7). ^‡ ^TB high burden countries (according to WHO [1]): Morocco (n = 2), Philippines (n = 1), Bosnia and Herzegovina (n = 1). BCG = Bacillus Calmette-Guérin; TB = tuberculosis; TST = tuberculin skin test.

None of the contacts reported seropositivity for HIV. Hepatitis C virus infection and immunosuppressive treatment were reported by one single subject each. Neither smoking habits, alcohol consumption, comorbidity, travelling to TB high burden countries within the past 12 months nor the presence of unspecific symptoms was associated with the test results in univariate or multivariate analysis (data not shown).

### Exposure to the source case

The median cumulative exposure time was 60 min and ranged from 3 to 4000 min (67 h). Eighty two subjects (57.3%) had had close contact to the index case. These included four individuals (2.8%), who had been exposed for >40 hours (Table 1). The cumulative exposure time correlated well with close contact to the index case (r = 0.54, p < 0.001).

### Interferon-γ release assay results

**Figure 2:**
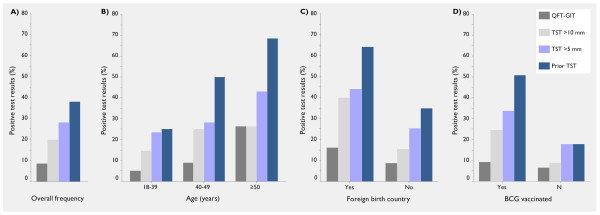
**Frequencies of positive test results**. Frequencies of recent positive test results (%) are displayed depending on: A) overall positivity; B) categorized age; C) birth in a foreign country; D) Bacillus Calmette-Guérin (BCG) vaccination. Prior TST results are plotted for comparison (dark blue column).

**Figure 3:**
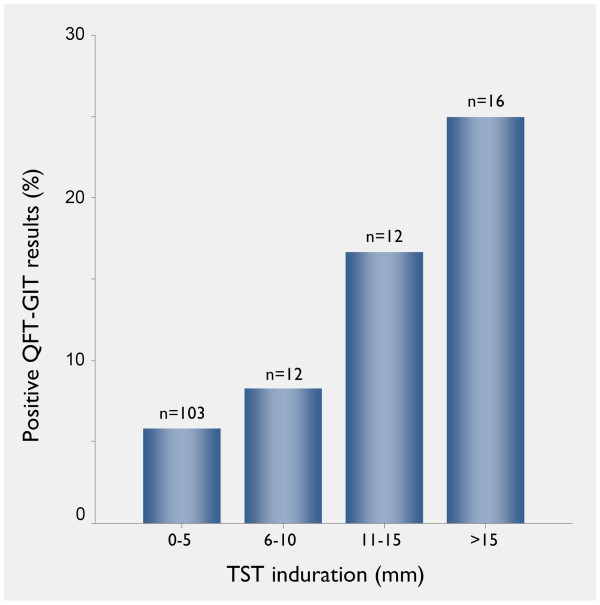
**Performance of the QFT-GIT in relation to Mantoux TST results**. QFT-GIT = QuantiFERON^®^-TB Gold in Tube; TST = tuberculin skin test.

QFT-GIT results were positive in 13 of the 143 contacts (9.1%). The QFT-GIT-positive subjects were significantly older (mean age [± standard deviation] 46 ± 10 vs. 37 ± 9 yrs, p = 0.006) and had been working in health care for a longer period of time than the QFT-GIT-negative subjects (mean 21 ± 12 vs. 12 ± 8 yrs, p = 0.032). However, there was no difference between median cumulative exposure times with regard to the QFT-GIT results (20 vs. 60 min, range 6 to 2625 min [44 h] vs. 3 to 4000 min [67 h], p = 0.31). Remarkably, the only subject with a history of prior TB in 1976 had a negative QFT-GIT (IFN 0.046 IU/ml), but a positive TST result (15 mm induration). Figure 2 shows positivity rates for the overall performance and the variables age (categorized), foreign origin and BCG vaccination status according to the diagnostic method and the TST cut-off applied. There was a trend towards higher QFT-GIT positivity rates with increasing TST induration (5.8%, 8.3%, 16.7% and 25.0% for induration categories 0–5 mm, 6–10 mm, 11–15 mm and >15 mm, respectively; p = 0.070; Figure 3).

### Tuberculin skin test results

The TST was read mean 72 ± 5 hours after application. Overall, 40 contacts (28.0%) and 28 contacts (19.6%) had a positive TST result when a cut-off >5 mm and >10 mm induration was applied, respectively. Mean age rather than mean duration of employment in health care, was significantly higher in TST-positive subjects when compared to TST-negative subjects (mean age 40 ± 9 vs. 36 ± 9 yrs and 41 ± 9 vs. 37 ± 10 yrs, p = 0.036 and 0.038, respectively; mean duration of employment in health care 15 ± 10 vs. 13 ± 8 yrs and 16 ± 10 vs. 13 ± 9 yrs, p = 0.25 and 0.14, respectively). Whichever TST cutoff was applied, there was no difference between the median cumulative exposure times (both 60 minutes, ranges 5 to 2520 min [42 h] vs. 3 to 4000 min [67 h], p = 0.48 and 0.85, respectively).

### Concordance between QFT-GIT and TST results and effect of BCG vaccination

More than one half of the contacts (51%) were BCG vaccinated (Table 1). Table 2 shows the agreement between QFT-GIT and TST results stratified according to the BCG vaccination status. The overall agreement between TST and QFT-GIT results was low when a cutoff >5 mm was applied and was only slightly higher for a cutoff >10 mm. With regard to those individuals who had not been BCG vaccinated, a better, but nevertheless low agreement was observed regardless of the applied cut-off. In total, concordant results between QFT-GIT and recent Mantoux TST results occurred in 72.7% of the subjects (104/143), predominantly in those with negative results in both tests (97/104, 93.3%) when a TST cutoff >5 mm was used. Discordant test results were observed in 27.3% of the subjects (39/143), most of them in the combination TST-positive/IGRA-negative (33/39, 84.6%; overall frequency 23.1%, 33/143), which was significantly associated with BCG vaccination (p = 0.020). An unknown BCG vaccination status was significantly associated with foreign origin (40% vs. 3.4% of subjects, p < 0.001). Data on BCG vaccination status was completely documented in individuals of Polish origin only (77.8% BCG vaccinated).

**Table 2:** Agreement between QFT-GIT and TST, stratified by BCG vaccination status

		QFT-GIT, n (%)		
		
	TST >5 mm	Positive	Negative	Agreement
All subjects	Positive	7 (4.9)	33 (23.1)	Raw = 72.7%
	Negative	6 (4.2)	97 (67.8)	**κ = 0.15**
BCG vaccinated	Positive	3 (4.1)	22 (30.1)	Raw = 64.4%
	Negative	4 (5.5)	44 (60.3)	κ = 0.04
No BCG	Positive	3 (5.4)	7 (12.5)	Raw = 85.8%
	Negative	1 (1.8)	45 (80.4)	**κ = 0.36**
				
	TST >10 mm	Positive	Negative	Agreement

All subjects	Positive	7 (4.9)	22 (15.4)	Raw = 79.7%
	Negative	6 (4.2)	108 (75.5)	**κ = 0.19**
BCG vaccinated	Positive	3 (4.1)	15 (20.5)	Raw = 74.0%
	Negative	4 (5.5)	51 (69.9)	κ = 0.12
No BCG	Positive	2 (3.6)	3 (5.4)	Raw = 91.1%
	Negative	2 (3.6)	49 (87.5)	**κ = 0.40**

Kappa (κ) values with statistically significant p values are printed **bold**. P values for TST >5 mm: All subjects, p = 0.048; BCG vaccinated, p = 0.69; no BCG, p = 0.016. P values for TST >10 mm: All subjects, p = 0.021; BCG vaccinated, p = 0.35; No BCG, p = 0.036. BCG = Bacillus Calmette-Guérin; QFT-GIT = QuantiFERON^®^-TB Gold in Tube; TST = tuberculin skin test.

### Comparison of current test results with prior TST results

One hundred and seventeen subjects (81.8%) had been tested with a prior TST median five years (range 3 mo to 38 yrs) ago. In most instances, prior TST had been administered by the multi-puncture method (92.3%, 108/117). Of those, 38.5% had had a positive prior TST result (Table 1). Positivity rates of prior TST results in relation to age, foreign origin and BCG vaccination status are shown in Figure 2 to provide a comparison with current test results. Prior TST results showed low overall agreement with recent Mantoux TST results (kappa = 0.38 and kappa = 0.32, p < 0.001 each, for an induration >5 mm and >10 mm, respectively), low overall agreement with QFT-GIT results (kappa = 0.09, p = 0.18) and low agreement with QFT-GIT even in non-BGC vaccinated subjects (kappa = 0.30, p = 0.077).

### Independent predictors of test positivity

Multiple logistic regression analysis confirmed the age dependency of positive QFT-GIT results (Table 3). The chance of having a positive QFT-GIT result increased about threefold with age (using three age categories, OR 2.7, 95% CI 1.32–5.46). However, no relation with BCG vaccination, foreign origin, exposure time per hour, close contact or any other variable was observed. Moreover, both foreign origin and BCG vaccination increased the probability of having a positive TST result about three- and fourfold depending on the respective cut-off applied. Again, no link to exposure (or family history of TB) was observed for the TST (Table 3).

**Table 3:** Multiple logistic regression analysis for positive TST and QFT-GIT results

	QFT-GIT ≥ 0.35 IU/ml	TST > 5 mm	TST > 10 mm
	
Variables	Adjusted OR (95% CI)	Adjusted OR (95% CI)	Adjusted OR (95% CI)
Male sex	1.0 (0.27–3.51)	1.1 (0.48–2.61)	1.5 (0.56–3.89)
Age categorized*	**2.7 (1.32–5.46)**^#^	1.6 (1.00–2.69)^#^	1.6 (0.90–2.82)^#^
Foreign birth country	2.5 (0.67–9.42)	**3.0 (1.03–8.99)**^#^	**4.4 (1.35–14.36)**^#^
BCG vaccination	1.7 (0.44–6.36)	**2.9 (1.19–6.86)**^#^	**4.2 (1.38–12.85)**^#^
Unknown BCG status	2.4 (0.36–16.20)	1.4 (0.31–6.32)^#^	2.6 (0.51–13.33)^#^
Exposure per hour	1.0 (0.95–1.07)	1.0 (0.93–1.02)	1.0 (0.95–1.04)
Close contact	0.7 (0.22–2.41)	1.0 (0.45–2.26)	2.0 (0.74–5.29)
Nursing profession/Physician	1.4 (0.44–4.75)	0.7 (0.31–1.54)	0.7 (0.30–1.83)
Affiliation with Pulmonary Care	0.8 (0.09–6.76)	0.5 (0.14–2.04)	0.5 (0.09–2.50)
Family history of TB	2.9 (0.46–18.01)	3.8 (0.83–17.63)	2.0 (0.40–10.16)

* Compare Table 1. ^# ^Variable included in final model building. OR and 95% CI with statistical significance are printed **bold**. BCG = Bacillus Calmette-Guérin; CI = confidence interval; OR = Odds ratio; QFT-GIT = QuantiFERON^®^-TB Gold in tube; TST = tuberculin skin test.

### Clinical impact of QFT-GIT test results and follow-up

Active TB was ruled out by physical examination and chest x-ray in all 13 participants with positive QFT-GIT results. Consultation and INH preventive therapy was offered to QFT-GIT-positive contacts only. Remarkably, only one QFT-GIT-positive HCW (7.7%) administered preventive therapy with INH as recommended. None of the contacts developed active TB within a period of two years (106 ± 1.5 weeks) after the last exposure to the index case.

## Discussion

The QFT-GIT proved to be feasible for contact tracing HCW in a low TB incidence in-hospital setting containing a high proportion of BCG vaccinated individuals even in a smear-negative index case. No secondary cases of active TB were detected within the observational period of two years, and the positive test results were not related to exposure. Altogether, relevant nosocomial TB transmission appears unlikely. The frequency of positive QFT-GIT results may in fact reflect the pre-existing prevalence of LTBI in the study population. Thus, IGRAs may offer the chance to increase the accuracy of diagnosing LTBI, enhance the implementation of preventive chemotherapy and further improve TB control in low-incidence countries and health care.

### Comparison with previous literature in the field

We determined a low overall frequency of positive QFT-GIT results of 9.1%. This frequency was substantially lower compared with the recent Mantoux TST (28.0%) or to the prior TST (38.5%). These findings are in agreement with studies on comparable populations determining the prevalence of LTBI among HCW without recent TB exposure. Just about one decade ago, Kralj and colleagues proposed a LTBI prevalence among German HCW of 40% according to positive multi-puncture TST results [19]. More recently, Nienhaus and Schablon and colleagues reported QFT-GIT positivity rates between 7.2–12.4% among German HCW [20-22]. In a Swiss study of HCW at a university hospital, a frequency of 7.6% was reported [23]. Similarly, Harada and colleagues concluded a LTBI prevalence of 9.9% among HCW in an intermediate-incidence country (Japan) using an earlier version of the QuantiFERON^®^-TB Gold assay [24]. A recent Australian study found results comparable to ours with regard to QFT-GIT and TST positivity (6.7% vs. 33.0%) and little agreement between both tests [25]. In contrast, three studies carried out among Japanese and Danish HCW and German radiologists detected even lower IGRA positivity rates of 3% and 1% respectively [26-28].

An informative comparison between the frequencies of positive IGRA results among the study population of HCW and the general German population is hampered by the lack of sufficient data on background IGRA positivity rates and the fact that the IGRA results depend to a great extent on the characteristics of exposure, the different settings and populations the test is applied to. Two recent contact studies that were conducted at an urban public health department among a population of non-HCW found QFT-GIT positivity rates of 10% and 11%, respectively, but included recent contacts of smear-positive index cases with extensive exposure >40 hours only [29,30]. Moreover, they contained a significantly higher proportion of foreign-born subjects of 27% and 30%, respectively, than observed in our study (18%). Another very recent IGRA contact investigation with a comparable epidemiologic setting and a major proportion of contacts of smear-negative source cases (48%) observed an overall QFT-GIT positivity rate of 24% (92/392) among the contacts of smear-negative source cases [31]. Remarkably, this study included contacts with positive TST results >5 mm induration only, more than half of the study population (52%) were foreign-born, and 55% of the contacts to smear-negative source cases had an aggregated exposure time >40 hours. However, the frequency of positive QFT-GIT results among the contacts of smear-negative source cases in the subgroup with an exposure time ≥ 40 hours was only 5% (9/176) compared to 9% (12/139) within the same subgroup in our study (data not shown). This observation may indicate a higher QFT-GIT positivity rate among HCW compared to the general population and may reflect an increased risk of TB infection among HCW [13,14].

Moreover, we found a low level of overall agreement between TST and QFT-GIT results. This finding is consistent with previous studies in HCW and thus confirms that the BCG vaccination is a major confounder of TST results, while QFT-GIT results were not affected by BCG [25,32,33]. Discordant results were frequently observed and occurred in 27.3% of the subjects with an overall frequency of 23.1% TST-positive/QFT-GIT-negative results. These findings support data provided by a recent meta-analysis that reported frequencies of 29.2% for overall discordant results and 24.1% for TST-positive/QFT-GIT-negative results, respectively [7].

Logistic regression analysis showed no obvious relation between exposure and positive results for either of the applied tests. Instead, we found age to be the only independent predictor of QFT-GIT positivity and demonstrated a further link between foreign origin, BCG vaccination and positive TST results. In previous contact tracing studies of profoundly contagious smear-positive pulmonary TB index cases, IGRA-positivity was well correlated with exposure [15,17,30]. In contrary, studies performed in low- and intermediate-incidence settings focusing on the prevalence of LTBI among HCW found age to be closely related to positive IGRA results [21,22,24].

We detected no secondary cases of active TB within a follow-up period of two years after the last exposure. Recently, first evidence for the relevance of positive QFT-GIT results was provided demonstrating a progression rate to active TB of 14.6% (6/41 subjects) over a two-year period in subjects who tested positive. However, this study only included subjects after recent exposure to smear-positive pulmonary TB >40 hours [30]. Another study found a progression rate of 8.1% (3/37) among HIV-1-infected subjects who were routinely screened for LTBI [34]. To date there are no studies available describing the predictive value of a single positive QFT-GIT result in absence of recent and profound smear-positive exposure or immunosuppression.

### Limitations

The present study is subject to limitations. Although only 82 HCW (57.3% of the study population, including four individuals with a cumulative exposure time >40 hours) had had close contact, all eligible subjects were included in this contact investigation contrary to current German and CDC guidelines and assigned to the medium to high priority category [6,35]. This pre-selection process may have reduced the pretest probability and subsequently the efficiency of the procedure. However, TB transmission is not necessarily correlated with the duration of contact, and the selection of contacts for screening should also be activity-based [36,37]. Nevertheless, given the unusual case presentation, the availability of sufficient resources and sparse evidence about the performance of IGRAs in the low-incidence in-hospital setting, we chose to include all eligible contacts of the particular index case.

Moreover, as there is no gold standard for the diagnosis of LTBI, both IGRAs and TST tend to indicate the lasting immune response after exposure to MTB rather than prove a genuine TB infection [38]. Despite the IGRAs' excellent specificity, the sensitivity of both QFT-GIT and TST is suboptimal at around 70%, and none of these tests is able to sufficiently discriminate between active disease and latent infection or between a recently acquired and a prior latent infection [9,33]. Most studies included in a recent, comprehensive meta-analysis used active TB as a surrogate for the evaluation of sensitivity and specificity, although the phenomenon of anergy is well known in active TB [9]. Hence, the IGRA responses of patients with active disease may not be representative of the condition of LTBI as exemplified by our index patient, who had a clearly negative QFT-GIT result (IFN 0.189 IU/ml) whilst suffering from severe active TB. Furthermore, according to national guidelines, we chose to x-ray QFT-GIT-positive subjects only, although no data sufficiently proves the superiority of the QFT-GIT in respect of sensitivity for detecting LTBI or active TB. In fact, this limitation may be emphasized by the particular HCW with the documented history of TB, who had a negative QFT-GIT result but a positive TST. In this context it should be noted that IGRA-negative contacts progressing to active TB have been reported [39,40], and therefore negative IGRA results should be interpreted with some caution.

### Interpretation of findings

The finding of age-dependency of positive QFT-GIT results may be due to an age-cohort effect based on steadily decreasing TB-incidence rates in Germany over the past decades and, on the other hand, to a longer time at risk whilst being employed in health care. This suggests that a significant proportion of the QFT-GIT-positive results were caused by prior MTB infection and not by recent exposure. Hence, our findings suggest a low contagiosity of the particular index case. Consequently, the frequency of positive QFT-GIT results may in fact reflect the pre-existing prevalence of LTBI among the study population and makes any relevant nosocomial transmission unlikely. The observed link between foreign origin and TST positivity may be due to the proportion of subjects with unknown BCG vaccination status among the subgroup of foreign born subjects, and may indicate substantially different BCG vaccination policies among countries in the past, as documented for Europe [41].

### Clinical relevance of findings

The QFT-GIT proved to be a feasible method in this large-scale, in-hospital contact investigation. Substantially lower prevalence rates of presumed LTBI resulted when different approaches of conducting contact investigations were employed, particularly compared with those that had been applied in the past (57.3%, 38.5%, 28.0%, 19.6% and 9.1% for a classification by close contact resulting in chest x-ray, prior TST, recent Mantoux TST with indurations >5 mm and >10 mm and QFT-GIT, respectively). This indicates that IGRAs have the potential to profoundly change our clinical practice. The high frequency of discordant results observed in our study argues against a two-step screening procedure in a low-incidence country with a substantial proportion of BCG vaccinated subjects. Moreover, our results support a recent study by Diel and colleagues, who suggested the feasibility of IGRAs in contact investigations of smear-negative index cases and, in this context, an exposure-dependent performance with markedly increased positivity rates only after exposure >40 hours [31]. Finally, since the consequently lower number of positive IGRA results offer the hypothetical chance to target preventive therapy, we will need to increase the poor acceptance of preventive therapy apparent in our study. In need of striking arguments, further research is necessary on the performance and predictive values of IGRAs in different settings and populations and on their dynamics over time [39,42].

## Conclusion

We did not detect any secondary case of active TB within the observational period of two years. Overall, the probability of relevant nosocomial transmission for the particular index case appears to be low. Our findings suggest that contact tracing is not generally warranted after cumulative exposure <40 hours if the index case is smear-negative. However, given the sensitivities of current IGRAs, they may not be used to sufficiently rule out the presence of LTBI. So far, no conclusive statement regarding the progression risk to active disease in our population and particular setting can be made. Both IGRAs and TST possess inherent limitations, and lack the ability to reliably discriminate between recently acquired or prior latent TB infection. Depending on the applied method, the prevalence of LTBI among the study population varied considerably. However, the substantially lower frequency of positive QFT-GIT results may provide the opportunity to target preventive therapy and thus contribute to enhanced TB control in health care.

## Competing interests

The authors declare that they have no competing interests.

## Authors' contributions

FCR conceived and designed the study, took care of adequate funding and equipment, performed the statistical analysis, took some blood samples, conducted and interpreted the ELISAs, interpreted the data, supervised the study and drafted the manuscript. SS participated in the study design, interviewed the HCW, applied and read the TST. AN participated in the study design, data interpretation, statistical analysis and revised the manuscript critically for important intellectual content. AS participated in the study design, data interpretation, statistical analysis and revised the manuscript critically for important intellectual content. GSW contributed to the study design and supervised the study. GR contributed to the study design, the analysis and interpretation of data, supervised the study and revised the manuscript critically for important intellectual content. All authors read and approved the final manuscript.

## Authors' information

Part of the data was presented at the 18^th ^European Respiratory Society Annual Congress 2008 in Berlin, Germany [43]. The site of the present study, the University Hospital Bergmannsheil, is an academic center for occupational diseases. It was founded in 1890 as the world's first Accident Hospital serving the coal mining population during industrialization.

## Supplementary Material

Additional file 1 **Definition of the index case**. The data provide radiological and microbiological details of the index patient Click here for file

Additional file 2 **Addendum methods section**. The data provide details of the QFT-GIT processing and the questionnaire items Click here for file

Additional file 3 **Detailed description of the subject with indeterminate QFT-GIT result**. The data provide clinical details of the subject with indeterminate IGRA result Click here for file
