# Supplementary material for: In-hospital contact investigation among health care workers after exposure to smear-negative tuberculosis
Source: J Occup Med Toxicol. 2009 Jun 8;4:11. doi: 10.1186/1745-6673-4-11 (PMC2698921; doi:10.1186/1745-6673-4-11)
Supplement: Additional file 1 — Definition of the index case. The data provide radiological and microbiological details of the index patient [file 1745-6673-4-11-S1.pdf]

In-hospital contact investigation among health care workers after exposure to smear-negative tuberculosis

Felix C. Ringshausen, Stephan Schlösser, Albert Nienhaus, Anja Schablon,

Gerhard Schultze-Werninghaus, Gernot Rohde

Additional file 1 – Definition of the index case

The index patient suffered from extensive smear-negative, culture-positive TB including non-cavitary pulmonary involvement. Briefly, the index patient had mistakenly received spinal irradiation at an external hospital for suspected metastatic lung cancer (in the presence of a compression fracture of the third thoracic vertebra and a coexisting solitary pulmonary node [tuberculoma] of the left upper lobe). Subsequently, his spinal TB (Pott's disease) flared up before being diagnosed and culturally confirmed at our institution after a complicated in-hospital course of about three months. Smear-negative specimen obtained from respiratory secretion, cerebrospinal fluid and the aspirate of a paravertebral abscess grew completely susceptible MTB. At that time, the patient was coughing and a CT scan of the chest detected multiple bilateral opacities with a tree-in-bud sign. Overall, this immobilized and dependent TB index patient had numerous in-hospital contacts in various medical departments and disciplines (Figure 1).
