# Supplementary material for: In-hospital contact investigation among health care workers after exposure to smear-negative tuberculosis
Source: J Occup Med Toxicol. 2009 Jun 8;4:11. doi: 10.1186/1745-6673-4-11 (PMC2698921; doi:10.1186/1745-6673-4-11)
Supplement: Additional file 2 — Addendum methods section. The data provide details of the QFT-GIT processing and the questionnaire items [file 1745-6673-4-11-S2.pdf]

In-hospital contact investigation among health care workers after exposure to smear-negative tuberculosis

Felix C. Ringshausen, Stephan Schlösser, Albert Nienhaus, Anja Schablon,

Gerhard Schultze-Werninghaus, Gernot Rohde

Additional file 2 – Addendum methods section

IGRA performance

Immediately after TST application, venous blood was collected from the study subjects into each of the three evacuated and heparinized collection tubes calibrated to draw 1 ml of blood. This whole blood assay uses two different peptides from the region of difference 1 (RD1) of the MTB genome (ESAT-6, CFP-10) and the peptide TB7.7 as specific antigens. Stimulation of IFN-γ-producing T-cells occurs within the collection tube, which is coated with the antigen mixture described above, when incubated at 37°C for 16 to 20 hours. Nil serves as negative, phytohemaglutinin (mitogen) as positive control. After centrifugation for 15 minutes at 3,000 rpm, IFN-γ release was promptly measured by enzyme-linked-immunosorbent-assay (ELISA).

Questionnaire items

Age, gender, department affiliation, profession, duration of service in health care, BCG vaccination status, date and results of prior multi-puncture or Mantoux TST, country of birth, smoking habits, alcohol consumption, HIV and hepatitis status, comorbidities, intake of immunosuppressive drugs, own, family (or recreational) history of previous TB (exposure), travelling to TB high burden countries for more than two weeks within the previous 12 months, presence of TB-related symptoms, average duration of exposure, frequency, intensity and time of exposure, chest radiographic findings and implementation of preventive INH chemotherapy, if applicable.
