# Supplementary material for: In-hospital contact investigation among health care workers after exposure to smear-negative tuberculosis
Source: J Occup Med Toxicol. 2009 Jun 8;4:11. doi: 10.1186/1745-6673-4-11 (PMC2698921; doi:10.1186/1745-6673-4-11)
Supplement: Additional file 3 — Detailed description of the subject with indeterminate QFT-GIT result. The data provide clinical details of the subject with indeterminate IGRA result [file 1745-6673-4-11-S3.pdf]

In-hospital contact investigation among health care workers after exposure to smear-negative tuberculosis

Felix C. Ringshausen, Stephan Schlösser, Albert Nienhaus, Anja Schablon,

Gerhard Schultze-Werninghaus, Gernot Rohde

Additional file 3 – Detailed description of the subject with indeterminate QFT-GIT result

The single subject with an indeterminate QFT-GIT result was a 26 year-old male physician at the department of surgery. He was working in health care for two years. He was born in Germany, had been BCG-vaccinated, and never had a TST before the recruitment TST, which was clearly negative without any induration. He never smoked, consumed alcohol rarely, had a documented negative HIV and hepatitis status, and did not report any comorbidity, intake of immunosuppressive drugs, own or family history of previous TB or previous TB exposure. He had not been travelling within the past 12 months and did not complain symptoms of active TB. He had a cumulative exposure time to the index case of 315 min (5.25 h) during seven consecutive weeks with an average contact time of eight minutes per contact and reported close contact to the index case within repeated physical examinations.

The QFT-GIT result was indeterminate due to a nil control >8.0 IU/ml. When the subject was retested the QFT-GIT result remained indeterminate, this time due to an insufficient mitogen positive control (minus nil control) <0.5 IU/ml.
